# Optimizing the energy bandwidth for transmission full-field X-ray microscopy experiments

**DOI:** 10.1107/S1600577521011206

**Published:** 2022-01-01

**Authors:** Malte Storm, Florian Döring, Shashidhara Marathe, Silvia Cipiccia, Christian David, Christoph Rau

**Affiliations:** a Diamond Light Source Ltd, Didcot OX11 0DE, United Kingdom; b Paul Scherrer Institut, Forschungsstrasse 111, 5232 Villigen PSI, Switzerland

**Keywords:** full-field microscopy, multilayer monochromator, energy bandwidth, X-ray imaging, transmission X-ray microscope

## Abstract

Full-field transmission X-ray microscopy (TXM) is a very potent high-resolution X-ray imaging technique. However, it is challenging to achieve fast acquisitions because of the limited efficiency of the optics. Using a broader energy bandwidth, for example using a multilayer monochromator, directly increases the flux in the experiment. The advantage of more counts needs to be weighed against a deterioration in achievable resolution because focusing optics show chromatic aberrations. This study presents theoretical considerations of how much the resolution is affected by an increase in bandwidth as well as measurements at different energy bandwidths (Δ*E*/*E* = 0.013%, 0.27%, 0.63%) and the impact on achievable resolution. It is shown that using a multilayer monochromator instead of a classical silicon double-crystal monochromator can increase the flux by an order of magnitude with only a limited effect on the resolution.

## Abstract 2

Results are presented for full-field transmission X-ray microscopy (TXM) acquired at different energy bandwidths; the trade-offs in achievable resolution and acquisition speed are discussed.

## 1. Introduction

Full-field transmission X-ray microscopy (TXM) is an established modality for nano-tomography measurements as it can offer short acquisition times and large fields of view which make it interesting for *in situ* applications which rely on fast measurements. It is widely used in many disciplines across fields like material science (Yuan *et al.*, 2016 ▸, 2018 ▸; Larsson *et al.*, 2019 ▸), biology and medicine (Mizutani *et al.*, 2019 ▸); acquisition times for full tomographies are typically around 10–60 min and have been improved towards 1 min (Ge *et al.*, 2018 ▸; Flenner *et al.*, 2020 ▸). The limiting factor in acquisition speed is the signal-to-noise ratio which should be larger than 5 (Rose criterion) and which is directly linked to the detector statistics. Improvements in acquisition speed can be achieved by either increasing the number of photons or by detector developments.

Common TXM setups still require relatively small effective pixel sizes on the detector because the achievable X-ray magnification is typically limited by the working distance required to be compatible with *in situ* environments. This fact prevents most beamlines from using efficient photon counting detectors and they must rely on scintillator-based detector systems combined with a CMOS or CCD chip. As scintillators emit over the full solid angle, only a fraction of the emitted photons can be collected in the camera system which makes these systems very photon inefficient.

The prevalent type of X-ray optics used in TXM experiments are Fresnel zone plates (FZPs) which are intrinsically inefficient and only diffract about 10–20% into the first order used for imaging. Blazed FZPs can be more efficient but are difficult to manufacture and not readily available with sufficiently small outermost zone widths for high-resolution TXM experiments (Di Fabrizio *et al.*, 1999 ▸; Mohacsi *et al.*, 2016 ▸). These two technical challenges limit the number of detected photons and make most TXM experiments statistics limited. Improving results requires longer exposure times or more photons. The time scale available for measurements is usually limited by the thermal stability, sample drifts and, in the case of *in situ* experiment, the speed at which the investigated process develops.

The acceptable energy bandwidth to reach the diffraction-limited resolution is equal to the inverse number of zones Δ*E*/*E* = 1/*N* with typical numbers of zones for hard X-ray FZPs in the range of a few hundred to a thousand. The question now is how much the resolution will be affected if this limit is breached. Typical energy bandwidths of multilayer monochromators (MLMs) are about or just beyond the limit defined by 1/*N*. The usual configuration is a Bragg crystal monochromator which has a very small energy bandwidth of Δ*E*/*E* ≃ 2 × 10^−4^. As the available energy bandwidth of synchrotron beamline sources – both undulators with harmonic peaks and bending magnets or wigglers with a continuous spectrum – is usually much broader, increasing the energy bandwidth translates directly into an increase in available photons. An MLM system has a much larger energy bandpass which – depending on the layout of the multilayers – can be between 0.1 and 4% (Morawe, 2019 ▸; Rack *et al.*, 2010 ▸; Kazimirov *et al.*, 2006 ▸). While commonly used for microtomography experiments, the use for TXM experiments is limited, also because the exact effect of the energy bandwidth on the resolution has not so far been systematically investigated. Since many TXM experiments are situated at beamlines which also offer microtomography and which are equipped with MLMs for microtomography, the widespread use of MLMs for TXM could improve throughput and statistics for many experiments where a modestly compromised resolution can be tolerated.

## 2. Fresnel zone plate depth of focus considerations

In the literature, the well established relationship between energy bandwidth and FZP parameters, most commonly given as number of zones *N*, is often given as a constraint to achieve diffraction-limited optical performance but little thought is given to how the system behaves if this limit is breached. Using a larger energy bandwidth will detrimentally affect the optical performance, but that might be an experimentally acceptable trade-off to achieve a higher flux.

In the following, we consider what happens when an FZP is illuminated with a broader energy spectrum.

First, we need to revisit some well established relationships for FZPs. Let us assume we have an FZP with a diameter *D* and an outermost zone width 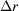 and are working at a wavelength λ. The number of zones is geometrically linked to these quantities by 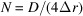 and the focal length is given by 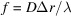 (see, for example, Attwood, 1999 ▸). The numerical aperture of a zone plate is given by 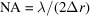 .

In the literature, the depth of focus (DOF) for a zone-plate-based microscope is usually defined by using the 20% decrease in intensity criterion to define the depth of focus (Attwood, 1999 ▸; Born & Wolf, 2019*b* ▸):

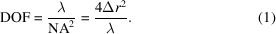

For FZPs in the hard X-ray regime, this number is typically on the order of below 100 µm (with focal lengths on the order of tens of mm).

Rearranging equation (1) and using the Rayleigh criterion for the resolution 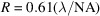 , it is

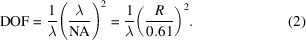

This formula gives a direct link between the achievable resolution and the depth of focus.

The relative focal length of the FZP changes proportionally to the relative change in the wavelength 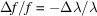 and it is 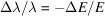 . Therefore, the shift of the focal length 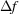 is directly proportional to the energy bandwidth.

If we assume that the depth of focus is limited by an energy bandwidth determined resolution limit 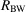 , the depth of focus of the optics will be larger than the change of the focal distance to allow all energies to be imaged in one plane at the highest resolution. Using 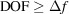 , and substituting, it is

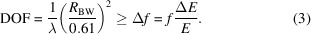

Solving this equation for 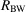 and substituting for the focal length, this yields

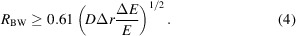

This is the bandwidth-limited resolution as a function of the energy bandwidth. As the diffraction limit holds as well, the best achievable resolution is limited by the larger of the two values:

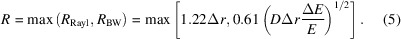




The point where the two expressions from equation (5) are equivalent gives the largest acceptable energy bandwidth without a deterioration in resolution. Solving this equality yields

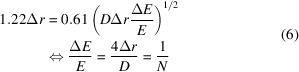

and gives the well described limit for the acceptable energy bandwidth. In addition, however, we can calculate a resolution limit for larger energy bandwidths.

**Figure 1:**
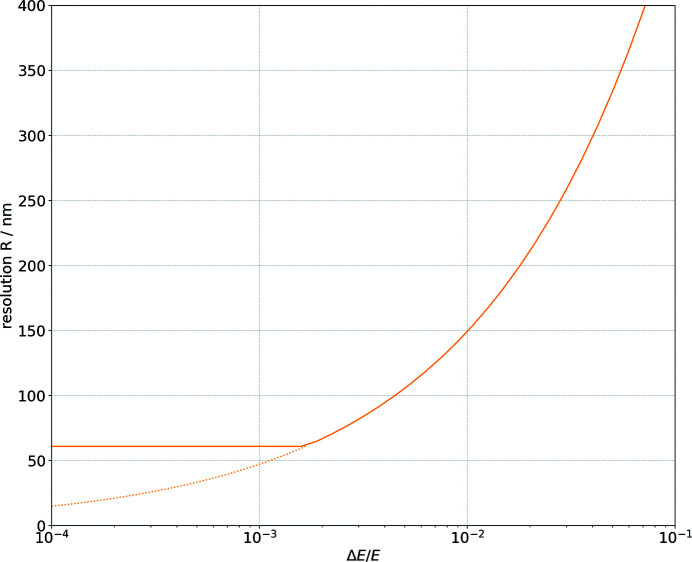
Plot of the resolution limit [as given by equation (5)] over the relative energy bandwidth. The achievable resolution deteriorates very quickly once beyond the acceptable 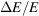 as outlined in equation (5). For 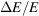 smaller than the Rayleigh criterion, the energy bandwidth limited resolution curve is continued as a dotted line. This example is calculated with *D* = 120 µm, Δ*r* = 50 nm, *N* = 600.

For *E* = 12 keV (λ = 0.103 nm), *D* = 120 µm and Δ*r* = 50 nm, this yields DOF = 97 µm. The acceptable energy bandwidth to stay within the DOF is Δ*E*/*E* = 1.7 × 10^−3^. This is almost an order of magnitude larger than the energy bandwidth of a Si-111 monochromator. The full plot of the achievable resolution for this example is given in Fig. 1 ▸. For example, for an FZP with *D* = 120 µm, Δ*r* = 50 nm, the limit given from equation (5) corresponds to Δ*E*/*E* = 1.7 × 10^−3^ whereas the theoretical energy bandwidth of a double-reflection Si-111 monochromator is Δ*E*/*E* = 1.3 × 10^−4^. Increasing the bandwidth even further will lead to a deterioration of the achievable resolution. However, that can be acceptable for some applications or if the experimentally achievable resolution is limited by factors other than the FZP, for example the NA of the illumination, the detector resolution, the mechanical stability of the setup or the statistics.

## 3. Experimental verification

### 3.1. Beamline layout

The effect of the energy bandwidth on the resolution has been investigated at beamline I13-2 at Diamond Light Source (Rau, Batey * et al.*, 2019 ▸; Rau, Storm *et al.*, 2019 ▸), which is equipped with a 2 m-long U22 in-vacuum undulator source with a minimal gap of 5 mm. The undulator yields a peak flux of 1.11 × 10^12^ photons mm^−1^ s^−1^ at 0.1% bandwidth at 12 keV in the optics hutch before filters and mirrors. A set of slits and a set of in-vacuum filters are installed in the beamline. For monochromatic operation, filters of 0.28 mm and 1.06 mm pyrolytic graphite are used to reduce the heat load on the downstream components.

A mirror with a fixed reflection angle (θ = 2.5 mrad) is installed in the imaging branch. This mirror has silicon, rhodium and platinum surfaces which allows different energy ranges to be selected with 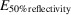 = 15.45, 32.9 and 41.4 keV, respectively, for the Si, Rh and Pt surfaces. The full reflectivity profiles are given in Fig. S1 (in the supporting information). While primarily used for tailoring the pink beam spectrum, the mirror also reduces the heat load on the monochromator and significantly helps in reducing higher harmonics.

The double MLM is outfitted with a Si substrate with three coatings: Ru/B_4_C, Mo/B_4_C and V/B_4_C (substrate by Carl Zeiss SMT GmbH, Oberkochen, Germany; coatings by Incoatec GmbH, Geesthacht, Germany). Key parameters for the multilayer stripes are given in Table 1 ▸. A simulation of the reflectivity curve at 12 keV has been obtained using the CXRO online multilayer reflectivity tool (Henke *Multilayer Reflectivity*, https://henke.lbl.gov/optical_constants/multi2.html) and is given in Fig. S2. The Ru/B_4_C multilayer system is designed for a very broad reflectivity whereas the Mo/B_4_C and V/B_4_C are designed for a much narrower energy bandwidth. The latter two are very similar because they are designed for different energy ranges. Note that the reflectivity curve of the Ru/B_4_C is much broader than an undulator harmonic and cannot be fully used. This is discussed in greater detail in the next section.

A second pair of slits is installed downstream of the MLM. In addition, a Si-111 double-crystal monochromator (DCM) in Bragg geometry is installed in the beamline. To allow an easy comparison with the multilayer systems, its key parameters are summarized in Table 1 ▸ as well. Both monochromators can be used individually or in combination.

### 3.2. Multilayer performance

**Figure 2:**
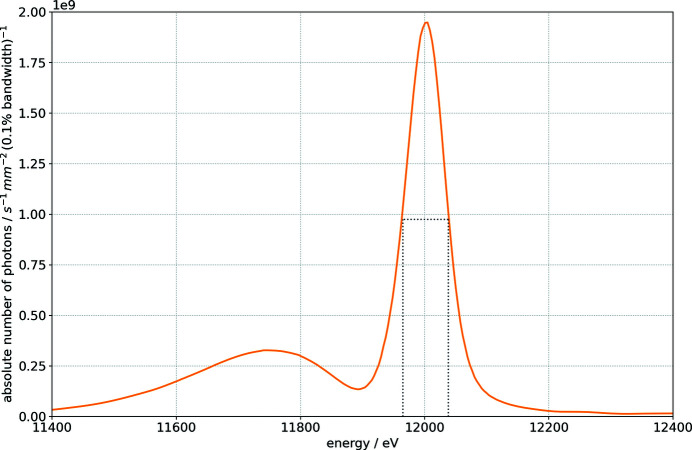
Measured absolute photon flux of the seventh harmonic peak of the undulator spectrum. The main peak has an FWHM of Δ*E* = 73.2 eV and the FWHM is shown as gray dashes. This curve is the envelope which determines the available energy bandwidth for the multilayer monochromator. The gap opening was 5.665 mm.

Details of the beamline energy calibration are given in Appendix *A* [App appa]. The MLM has been characterized to verify the reflectivity and energy bandwidth experimentally at 12 keV. The undulator gap has been set to an opening of 5.665 mm, which corresponds to the seventh harmonic. The spectrum has been measured by scanning the Si-111 DCM over the energy peak. The width of the main peak of the harmonic is Δ*E* = 73.2 eV or Δ*E*/*E* = 0.61%. A plot of the measured spectrum is given in Fig. 2 ▸.

**Figure 3:**
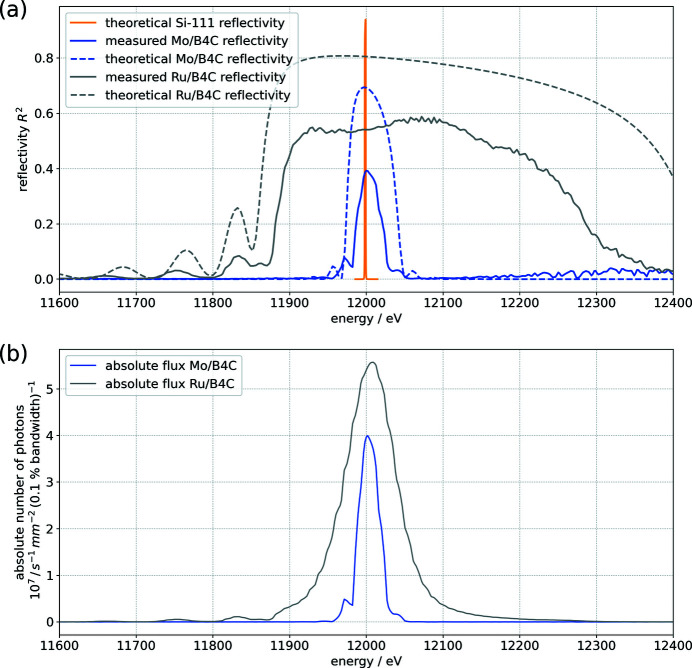
(*a*) Experimentally and theoretically determined reflectivity curves for double reflections of the Si-111 crystal and the Ru/B_4_C and Mo/B_4_C multilayer systems. (*b*) The flux measured at the experiment. This is the product of the monochromator reflectivity and the undulator harmonic photon flux.

The MLM reflectivity has been established by performing energy scans with the Si-111 DCM with and without the MLM in the beam for the Ru/B_4_C and Mo/B_4_C multilayer systems. The V/B_4_C multilayer system has not been investigated because it is intended for energies larger than 20 keV and the expected bandwidth is very similar to that of the Mo/B_4_C multilayer system. The detailed procedure is described in Appendix *B* [App appb] and the results are shown in Fig. 3 ▸(*a*) along with the simulation results. The experimentally measured performance is lower than the theoretical reflectivity but the curves show a good agreement. The lower reflectivity and energy bandwidth of the system could be caused by a lattice mismatch between the first and second crystal due to the different thermal load. The properties of the multilayer coating itself, like layer interdiffusion and inter-layer surface roughness, also affect both peak reflectivity and the energy bandwidth of the system. The absolute photon flux in the experimental hutch is shown in Fig. 3 ▸(*b*). The Ru/B_4_C curve is limited by the width of the undulator harmonic which is narrower than the Ru/B_4_C bandwidth. All the key numbers are given in Table 2 ▸. The effective accessible energy bandwidths in the experiment are thus 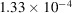 , 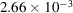 and 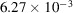 for the Si-111 DCM, Mo/B_4_C and Ru/B_4_C multilayers, respectively.

### 3.3. The I13-2 full-field X-ray microscope setup

The current TXM experiment is integrated in the microtomography setup of the I13-2 imaging branch and it is designed to operate in the energy range 8–15 keV (Storm *et al.*, 2020 ▸). These limits are primarily due to the efficiency of the optics (upper limit) and the beamline layout with windows and air paths (lower limit), which makes working outside of this energy range very inefficient.

The vacuum system exit window is installed 1.6 m from the sample position. A rotating decoherer can be installed here to reduce the partial coherence of the beamline even further. For these experiments, one layer of cooking parchment paper (density 40 g m^−2^) has been used. It is not strictly necessary to reduce the source coherence but to blur out distinct features in the illumination caused by filters, mirror and MLM which do move with monochromator vibrations and drifts.

**Figure 4:**
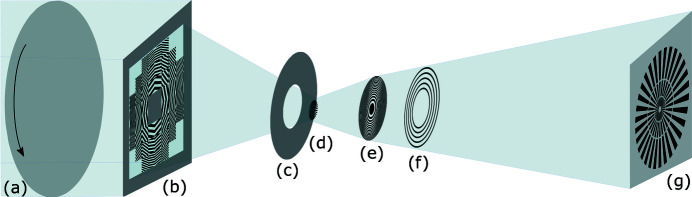
Sketch of the optical layout: (*a*) a rotating diffusor to reduce the coherence (optional); (*b*) a guard slit, central stop and beamshaping condenser: this ensemble provides the illumination. Note that the guard slits are only necessary because the beam size is larger than size of the order-sorting aperture. (*c*) The order-sorting aperture allows only the first diffraction order of the BSC to pass; (*d*) the sample is mounted in the working position of the optics; (*e*) the FZP focuses the magnified image of the sample on the detector system (*g*). Zernike phase rings (*f*) can be installed in the back-focal plane to change the contrast mechanism from absorption to phase contrast.

The condenser and central stop can be installed up to 1.5 m upstream from the sample and a vacuum pipe can be installed between the condenser and sample. An FZP acts as objective lens and Zernike phase rings can be installed for Zernike phase contrast (Schmahl *et al.*, 1995 ▸; Stampanoni *et al.*, 2010 ▸). A sketch of the X-ray optical layout is given in Fig. 4 ▸.

The rotation stage used for this experiment is a micos UPR-270 Air rotation stage and the sample is centered using an *xyz* assembly of three SmarAct SMC2430 positioners. All optical elements – condenser, central stop, order sorting aperture, FZP, Zernike phase ring – are mounted on *xyz* assemblies of SmarAct SMC2430 stages as well.

The experiment is installed in air and evacuated flight tubes are installed between the components. All components, except for the condenser assembly (including the central stop and decoherer) and detector, are mounted on a joint base to increase the relative stability and minimize drifts. A Hamamatsu C12849-101U camera with a 1:1 fiber-optic channel plate and a sCMOS chip with 6.5 µm pixel size is used as detector system. A slit system in front of the detector is used to limit the field of view to the area covered by the central stop.

All X-ray optics were designed and fabricated in the X-ray optics group at the PSI using electron beam lithography (Vila-Comamala *et al.*, 2011 ▸). A beamshaping condenser (BSC) with square fields (Vartiainen *et al.*, 2014 ▸) provides the illumination of the sample. It is designed to provide the same numerical aperture (NA) as the FZP and its layout is tailored to the Zernike phase rings, if installed. The FZP used for these experiments has a diameter of *D* = 90 µm and an outermost zone width Δ*r* = 50 nm. The acceptable Δ*E*/*E* to preserve the best resolution is Δ*E*/*E* = 2.2 × 10^−3^. The Zernike phase rings have a structure width *t* = 300 nm. The small width of the elements helps to suppress unwanted halo artifacts (Vartiainen *et al.*, 2014 ▸).

The full parameters of the optics are given in Table 3 ▸.

## 4. Results and discussion

A Siemens star test pattern (nominally 500 nm-thick gold layer on a 200 nm-thick silicon nitride membrane, manufactured by PSI Switzerland) with feature sizes down to 50 nm has been used to characterize the optical performance of the TXM with different monochromator bandwidths. The highest X-ray magnification feasible with the available X-ray optics and experimental setup has been used in this experiment. The effective pixel size on the camera has been determined to be 31.5 nm which corresponds to an X-ray magnification of *M* = 206.3×.

The detector point-spread function has been measured with a polished knife edge placed directly in front of the detector. The width of the knife edge profile between 10% and 90% intensity, which corresponds roughly to the Rayleigh criterion (Attwood, 1999 ▸), is 4.6 pixels. This corresponds well to the manufacturer’s specification of 33 line-pairs mm^−1^ which corresponds to 4.7 pixels. At an effective pixel size of 31.5 nm, the detector resolution limit is 144.9 nm full-period.

Projections of the Siemens star have been acquired with all three energy bandwidths and using both absorption and negative Zernike phase contrast. Using equation (5) to calculate the resolution limit for the setups used in this experiment yields resolution limits of *R*_Si-111_ = 61.0 nm, *R*_Mo/B4C_ = 66.7 nm, *R*_Ru/B4C_ = 102.4 nm. In theory, using the Mo/B_4_C multilayer has only very limited impact on the optical performance while the increased bandwidth of the Ru/B_4_C multilayer gives a significant deterioration of the resolution.

**Figure 5:**
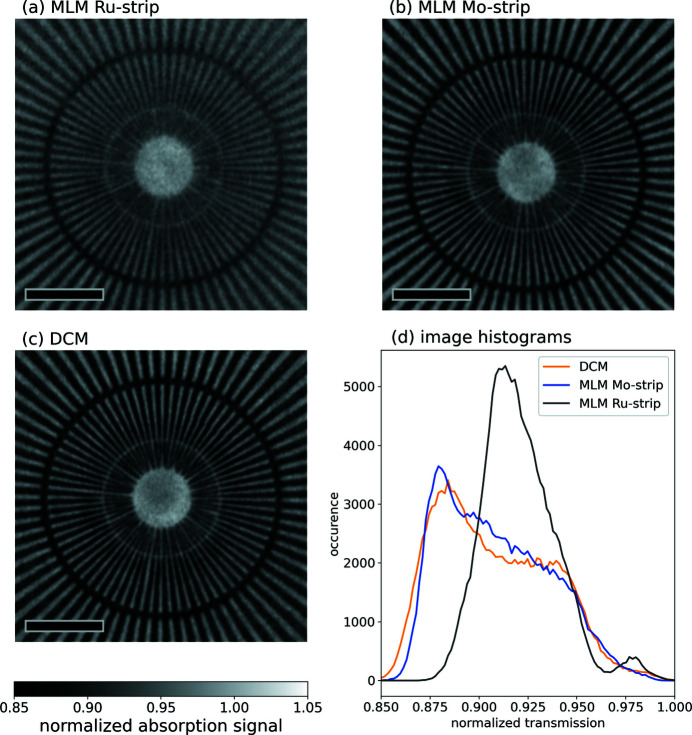
(*a*)–(*c*) Normalized (dark image and flat-field corrected) projections of the Siemens star in absorption contrast mode. The scale bar corresponds to 3 µm. The dark ring corresponds to 200 nm feature sizes, the light ring to 100 nm feature sizes and the smallest features on the Siemens star are 50 nm. (*d*) Histogram of the above images. The width of the main peak is equal for the Si-111 DCM and Mo/B_4_C MLM stripe and much narrower for the blurrier image acquired with the Ru/B_4_C MLM stripe.

**Figure 6:**
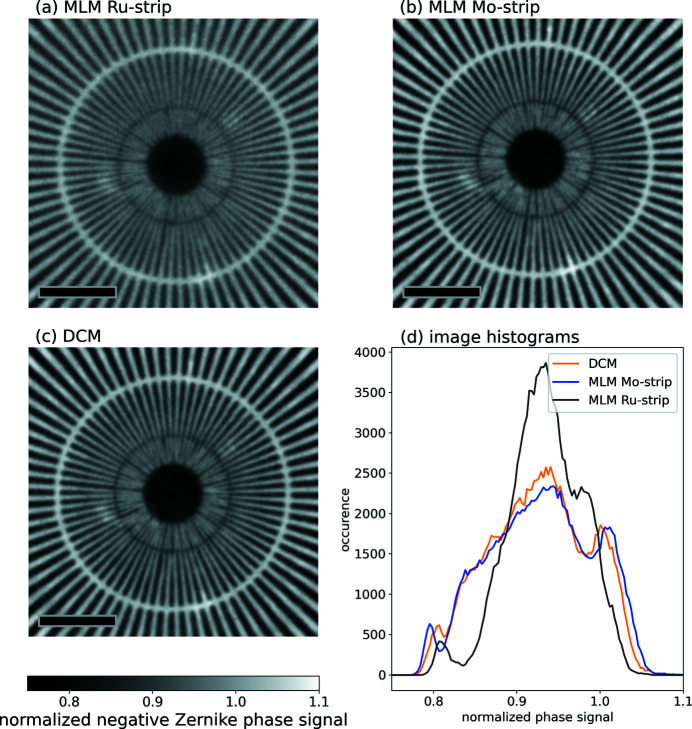
(*a*)–(*c*) Normalized (dark image and flat-field corrected) projections of the Siemens star acquired with the three different energy bandwidths in negative Zernike phase contrast mode. The black scale bar corresponds to 3 µm. The bright ring corresponds to 200 nm feature sizes, the dark ring to 100 nm feature sizes and the smallest features of the Siemens star are 50 nm. (*d*) Histograms of the above images. While the Si-111 DCM and Mo/B_4_C-MLM show a very similar behavior, the blurriness on the image for the Ru/B_4_C-MLM also shows in a reduced width of the histogram.

The absorption contrast images are given in Figs. 5 ▸(*a*)–5 ▸(*c*) and the corresponding figures for Zernike phase contrast are shown in Figs. 6 ▸(*a*)–6 ▸(*c*). Because the bandwidth and corresponding raw flux differ for the different monochromator settings, images have been acquired at different exposure times (details are supplied in Table S1). The figures clearly support the theoretical calculations and show very little difference in the results between the Si-111 and Mo/B_4_C images. In both cases, the resolution seems to be limited by the detector to about 100 nm feature size (corresponding to the innermost ring). The resolution for the Ru/B_4_C multilayer is limited to about 150 nm feature size and the image generally looks blurrier. It is also noteworthy that the general contrast level is significantly reduced for the Ru/B_4_C multilayer, as can be seen in the histograms in Figs. 5 ▸(*d*) and 6 ▸(*d*). The histograms for the two projections acquired with the Si-111 DCM and the Mo/B_4_C multilayer are similar, apart from statistical variations.

The resolution has also been quantified using Fourier ring correlation (FRC). Note that the actual field of view and image size are larger than shown in Figs. 5 ▸ and 6 ▸ and the Siemens star is well suited as test object to cover a wide range of frequencies. The resulting FRC curves have been smoothed with a Savitzky–Golay filter (Savitzky & Golay, 1964 ▸) before calculating the resolution. The resolution values have been calculated using the 1/2-bit resolution criterion (van Heel & Schatz, 2005 ▸) which is a more conservative measure than the 1/7 (Nieuwenhuizen *et al.*, 2013 ▸) commonly used in cryo-EM data. For this study, we are more interested in comparing different settings than achieving the lowest number. Also, the resolution determined by the 1/2-bit criterion matches quite well the resolution estimated from the visual inspection of the images. The influence of statistics on the FRC resolution has been investigated using a set of 20 projections and flat-fields acquired at one distance. Single images, the mean of two, four, six and ten images, have been used to calculate the resolution. For the case of ten averaged images, a random selection of ten out of 20 has been used (with the rest being used for the second image required for the FRC). In the case of less than ten averaged images, permutations of different images were processed to achieve about 150 different resolution values per averaging step. The full results are given in Table 4 ▸. The trend indicates that the achievable resolution, as determined by the FRC, is still limited by image statistics, *i.e.* the detector signal-to-noise ratio.

For each energy bandwidth, the sample was scanned through the FZP working position in steps of 5 µm to investigate the influence of the energy bandwidth on the resolution. The step width of 5 µm is small enough in comparison with the depth of focus of 48.3 µm to allow even small variations in the resolution to be picked up as one scans through the working position. A total of 20 projections was acquired at each position and two averaged images of ten projections each were used to determine the FRC resolution. The raw FRC results for varying focal positions have been fitted with second-order polynomials and the minima of these curves are used to determine the resolution limit. The numerical values are all listed in Table 5 ▸. The general trend can be well approximated with a second-order polynomial fit and the errors between data and fit are about two standard variations, as determined from the statistics test.

**Figure 7:**
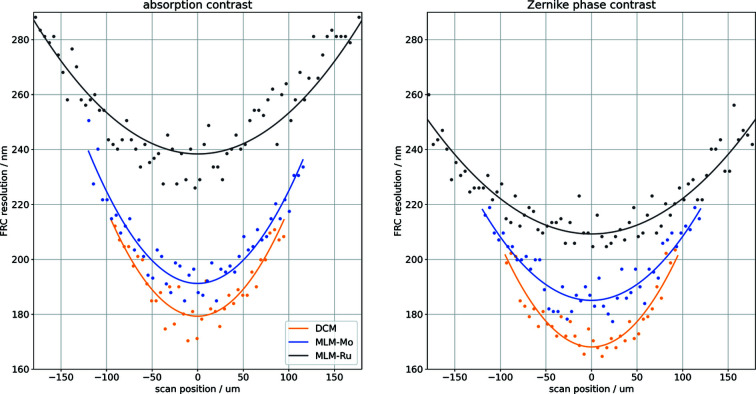
FRC results (1/2-bit criterion) calculated from the Siemens star test pattern for absorption contrast (left) and for negative Zernike phase contrast (right). The FRC shows that the resolution deteriorates with increasing energy bandwidth, as expected.

The resulting resolution values plotted over the sample position are given in Fig. 7 ▸. The FRC resolution for the different energy bandwidths follows the expected theoretical trend.

We expect two curves with only a slight offset for the Si-111 DCM and Mo/B_4_C multilayer and a larger offset for the Ru/B_4_C multilayer and we can observe this trend very clearly. As we aimed to keep statistics for each measurement comparable, we would have expected a similar offset between Si-111 DCM and Mo/B_4_C multilayer and Mo/B_4_C multilayer and Ru/B_4_C multilayer, respectively. For Zernike phase contrast, however, the calculated resolution for the Ru/B_4_C multilayer is much closer to the values of the other two bandwidths than expected.

**Figure 8:**
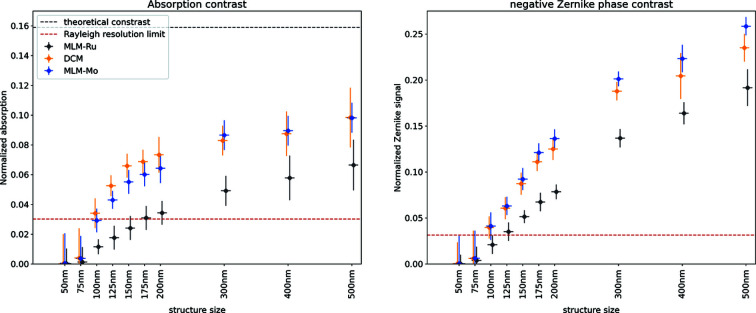
Contrast levels between line and space structures in the Siemens star for different structure sizes. The Rayleigh resolution limit, as defined by the X-ray optics, is plotted as well. For absorption (left), we have also given the theoretical contrast level (16% absorption). The data points for the Si-111 DCM are shifted very slightly to the left and the data points for the Ru/B_4_C MLM slightly to the right to improve the visualization, but the analysis has been performed for the same structure sizes. The legend applies to both subplots.

The nominal resolution is slightly better for Zernike phase contrast than for absorption contrast. Because the signal is stronger for Zernike phase contrast, the improved statistical definition of lines and spaces is expected to translate into a slight improvement in resolution as discussed in relation to the Rose criterion. In addition to the FRC calculations, the resolution has been calculated using the contrast level of the line/spaces in the Siemens star. An azimuthal integration at different distances from the center which correspond to line and space widths of 50, 75, 100, 125, 150, 175, 200, 300, 400 and 500 nm has been performed and the results are given in Fig. 8 ▸. For absorption contrast, the theoretical contrast level is known (transmission of 500 nm gold at *E* = 12 keV is *t* = 0.84) and the Rayleigh resolution limit is at a contrast of 0.19 of the normalized signal (Born & Wolf, 2019*a* ▸). For Zernike phase contrast, a similar value cannot easily be calculated as Zernike phase contrast is qualitative and not quantitative and this calculation is only performed for absorption contrast. However, the general form of the curves is very similar between absorption and Zernike phase contrast as expected.

**Figure 9:**
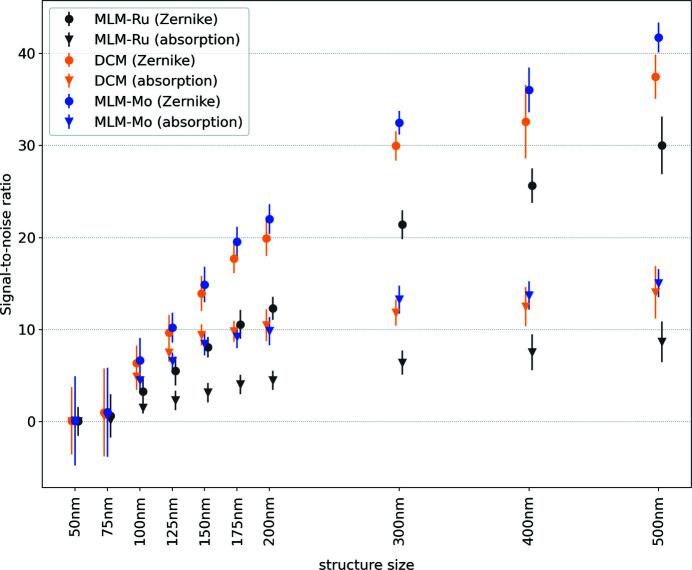
The signal-to-noise ratio for different structure sizes of the Siemens star for both absorption and Zernike phase contrast.

The resolution curves have been fitted with a function of the form

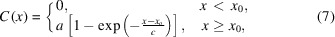

where *C*(*x*) is the contrast level, *a* is the amplitude and experimental limit, *c* is the time constant and describes how quickly the function approaches the limit and 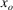 is an offset below which the contrast is zero. The full list of fitting parameters is given in Table S2 and the resolutions are given in Table 6 ▸. The ratio between the resolutions matches the theoretical expectations quite well even though there is an offset in the absolute resolution values. The results are consistent within themselves and the results for Si-111 DCM and Mo/B_4_C multilayer also compare well with the FRC results while the values for the Ru/B_4_C multilayer of 352 nm and 240 nm differ significantly. The larger energy bandwidth has a negative impact on the contrast level, as shown in the histograms in Figs. 5 ▸(*d*) and 6 ▸(*d*). While the FRC estimates the resolution from the sharpness of the edges, it does not take into account the contrast level as the Rayleigh criterion does and therefore yields a resolution which is better than expected for larger energy bandwidths where, in addition to the blurring, the contrast level has decreased as well. The benefit of using Zernike phase contrast is obvious if the signal-to-noise ratio (SNR) is considered. We define the SNR as

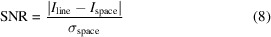

where *I*_line_ and *I*_space_ are the measured intensities for lines and spaces, respectively, and 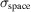 is the variation in the background. For practical reasons, 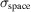 has been calculated using the empty region in the center of the Siemens star to exclude any effects from edges and halos. The SNR is plotted in Fig. 9 ▸. It is generally higher by about a factor of two for Zernike phase contrast. Also, the results of the Si-111 DCM and Mo/B_4_C multilayer are very close and indicate that an energy bandwidth only slightly larger than the FZP design limit is acceptable whereas a much larger energy bandwidth does lead to a loss of both resolution and contrast.

As the Siemens star is only a binary test pattern with high contrast, it might be argued that the effects of broadband illumination for objects of low contrast are more severe as changes to the contrast level have a stronger relative effect. While we agree that this effect could have implications for samples with lower contrast, this effect is very hard to quantify or model. Our experience at the beamline is that using the Mo/B_4_C multilayer in practice gives a good combination of statistics and resolution, even for samples with lower contrast like magnesium (Zeller-Plumhoff *et al.*, 2021 ▸) or shale (Wang *et al.*, 2021 ▸).

The increase in flux obtained with the larger energy bandwidth of the MLM may not be compatible with radiation-sensitive samples. The use of a condenser lens in TXM increases the flux on the sample. To give an example, a BSC with 3 mm diameter, field size of 60 µm, 10% diffraction efficiency into the first order, increases the flux by a factor of 196×. The measured flux density at 12 keV with the Mo/B_4_C multilayer is *N* = 4.82 × 10^11^ photons s^−1^ mm^−2^ in the experimental hutch. At 10% diffraction efficiency, this corresponds to a photon flux of 9.46 × 10^13^ photons s^−1^ mm^−2^ on the sample. So far, we have only seen issues with gas formation in liquids similar to those commonly experienced in microtomography or with biological specimen (insects). For radiation-sensitive sample systems, using the smaller bandwidth of the Si-111 DCM remains an option. Note, however, that the overall dose on the sample does not change with varying the bandwidth if the users aim for a similar count rate. In our experience, sometimes it is even advisable to measure faster with a higher energy bandwidth because some radiation damage effects occur rather slowly compared with the speed of measurements.

## 5. Conclusion

We have shown that TXM experiments are also possible with larger energy bandwidths than specified by the FZP resolution criterion and that the FZP resolution criterion of Δ*E*/*E* ≤ 1/*N* can be violated with only a moderate deterioration in experimental performance. Larger energy bandwidths have the advantage of higher flux and permit faster experiments. As a trade-off, the resolution and contrast levels drop if the energy bandwidth is increased too strongly. However, that might be an acceptable effect if it allows, for example, fast *in situ* applications with a moderate resolution.

For the specific case of I13-2, using the Mo/B_4_C multilayer yields an order of magnitude increase in flux while the resolution is reduced only marginally. The I13-2 TXM benefits from the fact that the experimentally determined energy bandwidth is about 40% smaller than the design parameters and that the reduced bandwidth only inflicts a minor resolution penalty. Using the Ru/B_4_C multilayer with its even larger bandwidth only yields an additional flux increase of approximately 3× over the Mo/B_4_C multilayer while the resolution deteriorates very strongly. The choice of an appropriate multilayer system with its energy bandwidth tailored to the TXM experiment’s parameters is paramount for optimizing both flux and resolution.

In addition, beamlines could explore the option of detuning double-reflection multilayer monochromators to tailor the bandwidth even further to experiments’ needs.

## Supplementary Material

Supporting figures and tables. DOI: 10.1107/S1600577521011206/ve5148sup1.pdf

## Figures and Tables

**Table 1:** Key parameters of the multilayer reflective coatings (for a single multilayer system) and the Si-111 reflection for comparison

	Ru/B_4_C	Mo/B_4_C	V/B_4_C	Si-111
*d* Spacing (Å)	46	20	25	3.1356
Number of layer pairs	100	400	400	
Gamma ratio	0.43	0.43	0.43	
Design energy range (keV)	6–22	6–20	20–39	
Theoretical peak reflectivity @ 12 keV	0.8078	0.6939	0.6000	0.969
Theoretical energy FWHM @ 12 keV (eV)	605.29	67.59	62.38	1.551
Relative energy bandwidth Δ*E*/*E* @ 12 keV	5.04%	0.56%	0.52%	1.3 \times {10^{ - 4}}

**Table 2:** Key photon flux and bandwidth parameters for the different experimental conditions The simulated FWHM is the product of the simulated monochromator reflectivity (squared for two crystals) and the simulated undulator spectrum. The measured FWHM for the monochromator only has been calculated by dividing the measurement with a reference measurement of undulator only.

	Undulator harmonic	Si-111 DCM	Ru/B_4_C MLM	Mo/B_4_C MLM
Simulated FWHM (eV)	85.1	1.6	84.2	51.2
Measured FHWM (monochromator only) (eV)	74.2	–	380.1	32.1
Measured FHWM (optics and undulator) (eV)	–	–	75.2	31.9

**Table 3:** The key parameters of the X-ray optics used for the energy bandwidth tests

Beam shaping condenser	Field size	60 µm × 60 µm
	Diameter *D*	2.9 mm
	Smallest structure size Δ*r*	50 nm
	Structure height *h*	1350 nm
Fresnel zone plate	Diameter *D*	90 µm
	Outermost zone width Δ*r*	50 nm
	Structure height *h*	950 nm
	Depth of focus at 12 keV	48.3 µm
	Working distance at 12 keV	43.8 mm
Phase rings	Phase ring width	300 nm
	Phase ring structure height *h*	1340 nm†
Detector	Working distance from sample	9.05 m

† This corresponds to a phase shift of π/2 at 12 keV.

**Table 4:** Calculated FRC resolution values for different numbers of averaged projections

Number of averaged projections	1	2	4	6	10
FRC resolution (nm)	262.1	233.1	214.4	205.7	186.0
Standard deviation (nm)	16.5	15.0	9.4	6.0	2.1
Difference between best and worst value (peak-to-peak) (nm)	70.9	61.4	35.1	17.2	11.2

**Table 5:** The calculated resolutions from the FRC for the different energy bandwidths and contrast modes The theoretical resolution limit has been calculated using the measured energy bandwidths discussed in the paper.

	Si-111 DCM absorption	Si-111 DCM Zernike	Mo/B_4_C MLM absorption	Mo/B_4_C MLM Zernike	Ru/B_4_C MLM absorption	Ru/B_4_C MLM Zernike
Theoretical resolution limit (nm)	61.0	61	66.7	66.7	102.5	102.5
Resolution limit normalized to Rayleigh limit	1.0	1.0	1.09	1.09	1.68	1.68
FRC full-period resolution (nm)	179.3	168.2	191.2	184.4	238.4	209.2
Resolution normalized to Si-111 DCM measurement	1.0	1.0	1.07	1.10	1.33	1.24
Standard variation fit data (nm)	4.87	4.13	4.95	4.72	7.33	5.19

**Table 6:** The resolution limit as determined from the azimuthal profiles The theoretical resolution limit has been calculated using the measured energy bandwidths discussed in the paper.

	Si-111 DCM absorption	Mo/B_4_C MLM absorption	Ru/B_4_C MLM absorption
Theoretical resolution limit (nm)	61.0	66.7	102.5
Resolution limit normalized to Rayleigh limit	1.0	1.09	1.68
Full-period resolution (nm)	191.4	207.2	351.8
Resolution normalized to Si-111 DCM resolution	1.0	1.08	1.84

## Appendix A Beamline energy calibration

The energy of the beamline has been calibrated using the Si-111 monochromator. The monochromator has been calibrated using the *K*1*s* absorption edges of copper, yttrium and molybdenum. The correct angle has been defined as the position when the absorption edge is centered in the field of view as there is a tiny vertical gradient caused by the source divergence. The error in the absolute calibration, as determined from the three data points, is Δθ = ±0.002°. At *E* = 12 keV, this corresponds to Δ*E* = ±2.50 eV.

## Appendix B Procedure for MLM reflectivity characterization

A calibrated photodiode (Canberra PD300-500 with light-tight foil, calibrated by Physikalisch-Technische Bundesanstalt, Berlin) was used to measure the total photon flux. The Bragg angle of the MLM and the pitch of the second MLM crystal were optimized to achieve the highest integral flux through a 3 mm × 3 mm slit system close to the experiment. Then, the Si-111 DCM was installed and a scan of the DCM Bragg angle was performed. The MLM was removed from the beam and the same DCM Bragg angle scan was repeated. The ratio of the two intensities, normalized to the electron beam current in the ring, gives the reflectivity of the double multilayer system.

For the Ru/B_4_C system, the reflectivity is much larger than the width of the undulator harmonic. For this case, the procedure described above has been performed for different positions of the undulator gap. The resulting individual scans have been stitched together to create a reflectivity curve with a larger energy bandwidth.
