# Supplementary material for: Optimizing the energy bandwidth for transmission full-field X-ray microscopy experiments
Source: J Synchrotron Radiat. 2022 Jan 1;29(Pt 1):138–47. doi: 10.1107/S1600577521011206 (PMC8733970; doi:10.1107/S1600577521011206)
Supplement: Supplementary file 1 [file s-29-00138-sup1.pdf]

JOURNAL OF SYNCHROTRON RADIATION

Volume 29 (2022)

Supporting information for article:

Optimizing the energy bandwidth for transmission full-field X-ray microscopy experiments

Malte Storm, Florian Döring, Shashidhara Marathe, Silvia Cipiccia, Christian David and Christoph Rau

Figure S1 Reflectivity curves for the I13-2 fixed-angle mirror ($\theta = 2.5mrad$). Orange: Silicon substrate; grey: ruthenium coating; blue: platinum coating

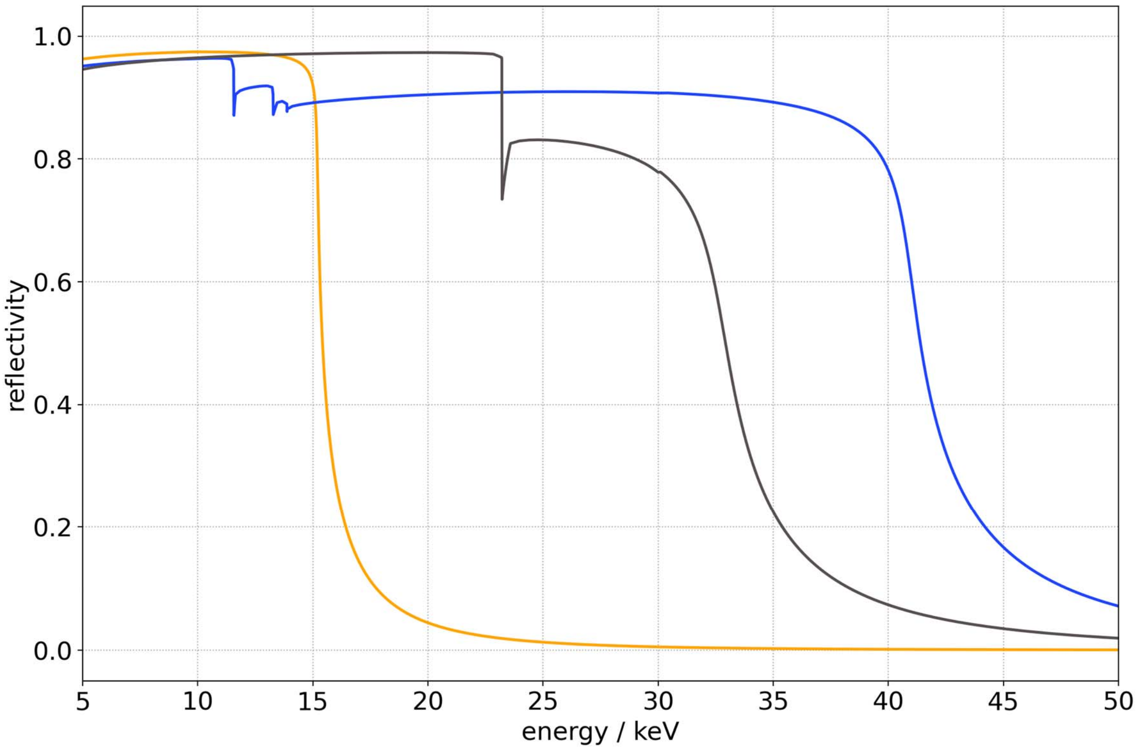

Figure S2 The theoretical reflectivity of the I13-2 multilayer systems at a nominal energy of 12 keV. The layer interdiffusion thickness assumed in the simulation 0.3 nm as per the manufacturer's specification. Orange: Ru/B_4C, blue: Mo/B_4C, grey: V/B_4C.

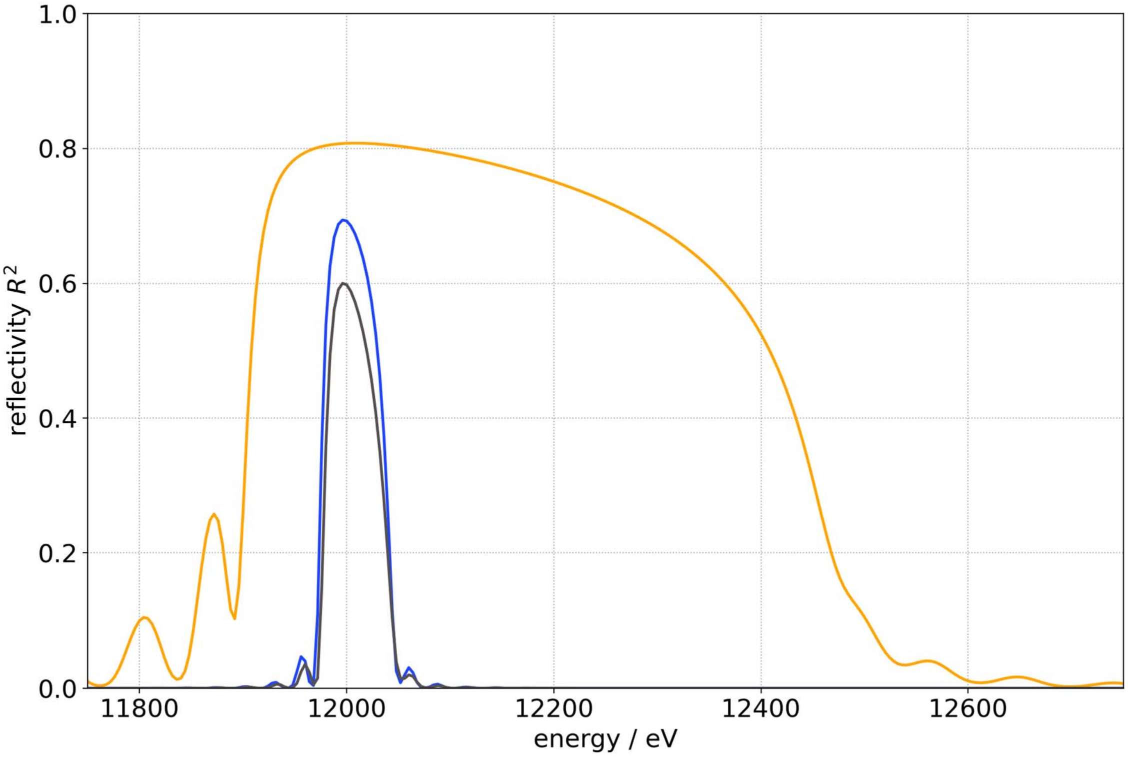

Table S1 Statistics and acquisition times used for the measurements of the Siemens star.

scan	Exposure time / s	Mean counts / pixel
Ru stripe, Zernike contrast	0.16	26066
Ru stripe, absorption	0.16	30606
Mo stripe, Zernike contrast	0.5	34470
Mo stripe, absorption	0.5	37663
Si-111, Zernike contrast	5.0	31633
Si-111, absorption	5.0	35219

Table S2 Fitting parameters for the spatial resolution determination from azimuthal profiles. The fit function is of the form $C(x) = a\left(1 - e^{-\frac{x-x_o}{c}}\right)$ for $x \geq x_0$.

	Si-111 DCM absorption	Si-111 DCM Zernike	Mo/B_4C MLM absorption	Mo/B_4C MLM Zernike	Ru/B_4C MLM absorption	Ru/B_4C MLM Zernike
a	0.0869	0.2642	0.0971	0.2886	0.0739	0.2901
x0	69.602	71.326	62.039	73.460	66.270	73.767
c	61.220	192.746	111.533	194.275	208.845	382.640
